# Exploring the Influence of Potential Entrepreneurs’ Personality Traits on Small Venture Creation: The Case of Saudi Arabia

**DOI:** 10.3389/fpsyg.2022.885980

**Published:** 2022-04-22

**Authors:** Ali Saleh Alshebami, Abdullah Hamoud Ali Seraj

**Affiliations:** ^1^Applied College, King Faisal University, Al Hofuf, Saudi Arabia; ^2^Department of Management, College of Business Administration, King Faisal University, Al Hofuf, Saudi Arabia

**Keywords:** SMEs, entrepreneurs, innovativeness, Saudi Arabia, internal locus of control

## Abstract

This study examined the impact of selected personality traits—innovativeness, internal locus of control, need for achievement and propensity to take risks—on the entrepreneurial intention of Saudi students (young entrepreneurs). The study sample included 165 students from an applied college affiliated with King Faisal University. The participants completed an online self-administered questionnaire, the data from which were analyzed using the partial least squares structural equation modeling (PLS-SEM) method. The findings revealed that the characteristics of innovativeness, internal locus of control and propensity to take risks had a positive relationship with entrepreneurial intention. However, the need for achievement had no relationship with entrepreneurial intention. The study model predicted approximately 25% of the total variance in entrepreneurial intention. It is recommended that in future studies, the sample size should be increased and the scope of the study should be broadened.

## Introduction

Entrepreneurship and small and medium-sized enterprises (SMEs), since their inception, have been considered key drivers for economic growth and development, the creation of new job opportunities, the mitigation of poverty, and the resolution of socioeconomic problems (Li et al., 2020; Yaser et al., 2020; Alshebami, 2021; Arkorful and Hilton, 2021; Cai et al., 2021; Chew et al., 2021; Elnadi and Gheith, 2021; Jiatong et al., 2021). Entrepreneurship is the process of identifying a business opportunity, pursuing the opportunity, and developing the necessary skills to maximize its benefits. Entrepreneurship is also the process of venture creation (Liñán and Chen, 2009). Entrepreneurship has received much attention recently by different scholars who have looked at it from different points of view.

The significance of entrepreneurship to economic growth and development has compelled governments in both developed and developing countries to design and implement the essential tools and support needed to stimulate entrepreneurial endeavors and encourage an entrepreneurial mindset in individuals. Various initiatives have been launched for this purpose and have largely been directed toward young individuals as they are the economic producers of the future (Bellò et al., 2018). Supporting entrepreneurship may include the provision of adequate entrepreneurship ecosystems capable of attracting entrepreneurs (Ali et al., 2019). In addition, the presence of stiff competition and various market challenges imposes ressures such that focusing on physical ecosystems alone may lead to the incomplete development of entrepreneurship and entrepreneurial intention. This is a particularly salient consideration given that entrepreneurial behavior is strongly predicated on entrepreneurial intention (Ajzen, 1991; Liñán and Chen, 2009), which is in turn influenced by personal traits or characteristics, including those of a psychological nature.

According to Koh (1996) and Gurol and Atsan (2006), internal locus of control, the need for achievement, innovativeness and the propensity to take risks are considered key to the development of entrepreneurial intention. To these, Bellò et al. (2018) added self-efficacy and the desire to innovate. In the extant literature, the need for achievement has been defined as the desire or determination to succeed in a competitive environment (Schaper et al., 2010). Meanwhile, innovativeness has been described as the ability to recognize and carry out entrepreneurial tasks in a creative manner (Nasip et al., 2017). Another key to entrepreneurial intention, the propensity to take risks, has been likened to capacity building because it promotes a positive attitude toward self-efficacy (Naushad, 2018). Finally, internal locus of control is regarded as feelings or conceptions about critical elements that affect or cause life events (Koh, 1996). These personal traits, despite their assumed importance, have received relatively little research attention with respect to their effect on entrepreneurial intention, particularly among young entrepreneurs in various parts of the world (Nasip et al., 2017; Bhatti et al., 2021).

In the case of Saudi Arabia, which is a developing country largely dependent on oil revenue, the steady decline in oil prices worldwide has led to a notable budget deficit and the consequent struggle to cover its expenses. Accordingly, the Saudi government developed a comprehensive plan, named “Saudi Vision 2030,” to introduce large-scale reforms to various economic sectors. Included in these reforms is heightened support for entrepreneurship and SMEs, some explicit aims of which are to increase the contribution of SMEs to the national GDP from 20 to 35%, reduce the unemployment rate from 12.9 to 7% by 2030, and encourage participation by young females in the labor market (Khan and Alsharif, 2019; Aljarodi, 2020; Aloulou, 2021; Alshebami and Seraj, 2021b). Another goal of the Saudi Vision 2030 plan is to create about 6 million job opportunities by 2030 (Elnadi and Gheith, 2021). Accordingly, the Saudi government and other official national entities have established various funding institutions, entrepreneurial initiatives, and incubators to promote the development of entrepreneurship and SMEs in the country (Khan and Alsharif, 2019; Alshebami et al., 2020).

According to Roomi et al. (2021), Saudi Arabia is currently positioned sixth in the Global Entrepreneurship Index (GEI) due in large part to the economic support provided by the government to improve the economy in general and to mitigate the economic effects of COVID-19 in particular, especially among SMEs. This support has been considered essential because the SME sector in Saudi Arabia includes approximately 99.6% of all private sector ventures.

Therefore, to further support the Saudi government’s efforts to erect institutional structures and cultivate entrepreneurship among citizens, young citizens in particular, to better facilitate the launching of small businesses, it is important to expand knowledge about the key personal traits that contribute to entrepreneurial intention, especially among younger adults (Nasip et al., 2017). In the Saudi context, for entrepreneurs to succeed, it is particularly essential to explore personal traits directly associated with business establishment (Bhatti et al., 2021). More specifically, it is vital to investigate levels of readiness among young Saudis in terms of their entrepreneurial intention to start small businesses. Likewise, it is crucial to identify which factors potentially motivate individuals to become entrepreneurs in the first place. Also of importance is to determine the pathways through which entrepreneurial intention among young graduates is stimulated (Shahzad et al., 2021).

In the extant literature on entrepreneurship in Saudi Arabia, it is noted that, despite the variety of existing studies, few examinations have been conducted on the personal traits responsible for the development of entrepreneurial intention. Instead, most research has focused on entrepreneurial education, psychological capital, formal institutions and related aspects (Aljaghthami and Noormala, 2016; Ibrahim and Amari, 2018; Alkahtani et al., 2020; Aloulou, 2021; Alshebami et al., 2020; Sharahiley, 2020; Yaser et al., 2020; Alshebami and Seraj, 2021a). Comparatively few studies have concentrated on psychological characteristics and their connections to other factors, such as training (Naushad, 2018; Bhatti et al., 2021).

Accordingly, and based on calls by previous studies to continue to explore the key personal traits responsible for the development of entrepreneurial intention among young adults, the present research investigated selected personal traits believed to influence entrepreneurial intention among students enrolled at an applied college affiliated with King Faisal University. It was considered important to address this population as they will be the drivers of the future economy of Saudi Arabia. Additionally, as students of applied colleges (which offer only diploma programs) are less likely than bachelor’s degree students from other colleges to find employment within the government sector, encouraging them to become entrepreneurs is a worthwhile pursuit. The following research question was formulated based on this underlying rationale:

1. Do internal locus of control, need for achievement, innovativeness and propensity to take risks enhance entrepreneurial intention among Saudi students at an applied college affiliated with King Faisal University?

This article is organized into the following sections. Following the introduction, the literature review and hypotheses are presented. Then, the methodology of the study and the analysis of the research results are described, after which these results and their implications are discussed. Lastly, some conclusions based on the study are provided.

## Literature Review and Hypothesis Development

### Personal Traits and Entrepreneurial Intention

Ajzen (1991) argued that intention was the best predictor of behavior. Going further, Ajzen claimed that intention is affected by three factors: attitude toward behavior, subjective norms and perceived behavioral control. Each of these factors can either positively or negatively impact entrepreneurial intention (Al-Jubari, 2019; Alshebami et al., 2020; Francisco Liñán and Chen, 2009). Entrepreneurial intention, the initial step in the entrepreneurial process (Elnadi and Gheith, 2021; Shahzad et al., 2021), can be defined as the willingness and desire to establish and own a business.

Entrepreneurial intention can be influenced by a variety of factors, such as institutional factors, or so-called ecosystem factors (Ali et al., 2019), or personal factors, such as internal locus of control, need for achievement, innovativeness and propensity to take risks (Koh, 1996; Nasip et al., 2017; Ndofirepi, 2020; Bhatti et al., 2021). Personal factors such as these can generate different and often conflicting results in diverse contexts. An important question, then, is why these personal factors cause some individuals to become entrepreneurs and not others (Nasip et al., 2017). To address this issue, we examined these personal traits among Saudi students at an applied college and their relation to entrepreneurial intention, borrowing from theories linking personality traits to entrepreneurship (Krueger, 2000).

### Innovativeness and Entrepreneurial Intention

Innovativeness is the quality of being or producing something novel, unique, remarkable or original (Staniewski et al., 2016). In the commercial domain, innovativeness can culminate in the establishment of start-ups that market original products or services and/or involve novel business activities or marketing approaches (Koh, 1996). Such ventures can make significant contributions to economic development and growth (Lewandowska et al., 2021). Innovativeness also plays a pivotal role in the cultivation of entrepreneurial intention in terms of conduct, attentiveness, and the use of technology to develop business models and strategies (Koe, 2016; Nasip et al., 2017; Wathanakom et al., 2020; Shahzad et al., 2021), as evidenced by the fact that entrepreneurs are typically more innovative than ordinary people (Robinson et al., 1991). Entrepreneurs rely on their innovative faculties to develop new products and services as well as to find solutions to challenging issues (Wathanakom et al., 2020). Accordingly, the following hypothesis was formulated:

H1: Innovativeness positively influences entrepreneurial intention among Saudi students.

### Internal Locus of Control and Entrepreneurial Intention

Conceptually speaking, the origin of locus of control can be traced to personality theory (Rotter, 1966). In practice, locus of control refers to feelings or perceptions regarding crucial elements that influence or cause life events. Two types of locus of control have been identified: internal and external. In this study, focus was placed on internal locus of control, which is the degree or extent to which people believe they possess the ability to control and manage their day-to-day lives (Arkorful and Hilton, 2021). Internal locus of control is believed to play a key role in the development of entrepreneurial intention. And yet, the extant literature on the relationship between internal locus of control and entrepreneurial intention has generated conflicting results (Rauch and Frese, 2007; Ferreira et al., 2012).

For example, Koh (1996), Mueller and Thomas (2000), and Gurol and Atsan (2006) demonstrated a positive connection between internal locus of control and entrepreneurial intention. More specifically, they found that students with a high internal locus of control had a stronger entrepreneurial intention. Other researchers, however, have found no such positive association between internal locus of control and entrepreneurial intention (Ferreira et al., 2012; Dinis et al., 2013). Still, it is reasonable to suspect that those who possess a high internal locus of control are more likely to become entrepreneurs (Lefcourt, 2014; Vodă and Florea, 2019; Arkorful and Hilton, 2021) and to more effectively find and implement solutions to related challenges (Kusumawijaya, 2019; Zhao and Wibowo, 2021). Accordingly, the following hypothesis was proposed:

H2: Internal locus of control positively influences entrepreneurial intention among Saudi students.

### Need for Achievement and Entrepreneurial Intention

McClelland (1961) theorized about the need for achievement, which can be defined as the desire to excel in competitive environments (Schaper et al., 2010). The need for achievement is believed to be a key personal trait that drives the behavior of individuals in general and entrepreneurs in particular (Koh, 1996). In the domain of entrepreneurship, the need for achievement is one of many factors responsible for motivating individuals to engage in venture creation (Shaver and Scott, 1992). Importantly, it has also been found that the need for achievement is an especially influential determinant for entrepreneurial intention among individuals in general and students in particular (Tong et al., 2011; Ferreira et al., 2012; Nasip et al., 2017; Kusumawijaya, 2019; Ndofirepi, 2020). In other words, those individuals with a greater need for achievement tend to have stronger entrepreneurial intention (Naushad, 2018) and to consequently take the actions needed to become entrepreneurs (Robinson et al., 1991). Therefore, the following hypothesis was developed:

H3: The need for achievement positively influences entrepreneurial intention among Saudi students.

### Propensity to Take Risks and Entrepreneurial Intention

The propensity to take risks also acts as a significant determinant of entrepreneurial intention. This propensity can be described as the degree to which one determines that the benefits of an action outweigh the risks. The propensity to take risks is often based on subjective risk assessments and interpretations (Marton et al., 2021). It is also implicated in capacity building since it instills in people a better attitude toward self-efficacy (Naushad, 2018) and as such makes them more likely to take risks (Koh, 1996). The extant literature has shown that the propensity to take risks influences entrepreneurial intention (Gurel et al., 2010; Uddin and Bose, 2012; Ndofirepi, 2020; Shahzad et al., 2021) and, furthermore, makes those with a greater propensity to take risks more competitive (Shahzad et al., 2021). Accordingly, the following hypothesis was formulated:

H4: The propensity to take risk positively influences entrepreneurial intention among Saudi *students.*

### Research Model

**FIGURE 1:**
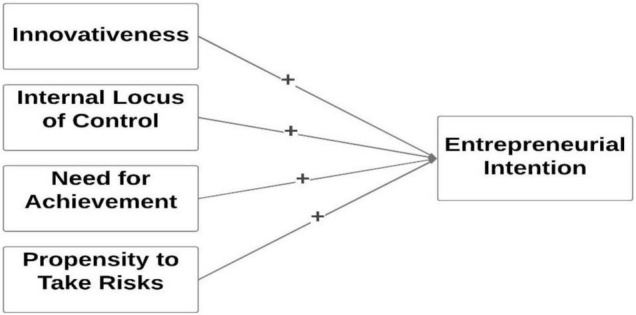
Conceptual model. Source: Author elaboration.

Figure 1 depicts the hypothesized model of the study.

## Research Methodology

### Participants and Procedures

The study sample was recruited *via* the administration of an online questionnaire to students of the Abqaiq applied college, which is affiliated with King Faisal University in Saudi Arabia. The college offers two diploma programs: a human resource management (HRM) program and a medical secretary program. The study sample comprised 165 participants, both male and female. These students were targeted because their diploma programs can be completed in a short duration of time and also because the students are expected to be potential entrepreneurs. The researchers adopted measures employed in previous studies and carefully translated them into the Arabic language. A pilot study was first conducted with 15 respondents to assess the validity of the questionnaire. As no validity concerns were raised, the questionnaire was administered to the students (potential entrepreneurs) in the main study, remaining online for 1 month. Table 1 gives a brief outline of the demographic information of the study participants.

**TABLE 1:** Demographic information.

	Range/Type	Frequency	(%)
Gender	Male	69	58.2
	Female	96	41.8
	Total	165	100
Age	18–27	165	100
Experience	Yes	48	29.1
	No	117	70.9
	Total	165	100
Program	HRM	162	98.2
	Medical secretary	3	1.8
	Total	165	100

*Source: Primary data.*

Table 1 show that male respondents made up 58.2 percent of the total, while female respondents made up 41.8 percent. The respondents ranged in age from 18 to 27. Furthermore, 70.9 percent of respondents stated that they have no prior experience managing a business. Furthermore, 98.2 percent of students are enrolled in the HRM program, while 1.8 percent are enrolled in the medical secretary program.

### Measures

In Table 2, the measures used in the study are listed.

**TABLE 2:** Measurement sources.

Construct	Source
Entrepreneurial intention	Liñán and Chen, 2009
Personal traits	Koh, 1996

*Source: Author elaboration.*

The items investigated in the study were measured using a 5-item scale, with 1 representing “total disagreement” and 5 representing “complete agreement.”

## Data Analysis and Interpretation

To analyze and interpret the research data, two steps were required: evaluating the measurement model, and evaluating the structural model.

### The Measurement Model

In the measurement model, the reliability and convergent validity of the measures used in the study were examined carefully. First, the indicator loadings were evaluated. In this test, the loading value was recommended to be 0.70 or above. If so, then the measured construct could be determined to explain 50% of the variance in the indicator, thereby demonstrating acceptable reliability (Hair et al., 2019). However, the decision to remove items with loading values below 0.70 should depend on whether doing so would increase composite reliability. That said, items with loading values below 0.40 should definitely be removed (Hair et al., 2011, 2017).

The second step in the evaluation of the measurement model included the assessment of the reliability of internal consistency using the composite reliability test. In this test, the higher the composite reliability values, the greater the reliability. A composite reliability value between 60 and 70 is considered acceptable (Hair et al., 2017). The third step involved the evaluation of convergent validity, which is the degree to which a measure compares favorably with another measure of the same construct. In this step, average variance extracted (AVE) was used for this purpose. The recommended value of the AVE should be 50% or higher, as this indicates the ability of the constructs to explain more than 50% of the variance of the indicator (Hair et al., 2011, 2019). Table 3 presents the findings of the indicator factor loadings, composite reliability and AVE.

**TABLE 3:** Reliability and convergent validity.

Construct	Loadings	Composite reliability	Average variance extracted (AVE)
Entrepreneurial intention		0.904	0.702
EI3	0.821		
EI4	0.815		
EI5	0.835		
EI6	0.880		
Innovativeness		0.781	0.543
Inov2	0.708		
Inov3	0.778		
Inov4	0.723		
Internal locus of control		0.790	0.653
LOC4	0.841		
LOC6	0.774		
Need for achievement		0.814	0.686
NFA1	0.813		
NFA5	0.843		
Propensity to take risks			
PTTR2	0.863	0.814	0.687
PTTR3	0.793		

*Source: Primary data.*

Table 3 shows that all results aligned with the recommended values such that their validity and reliability were satisfactory.

The fourth step in the evaluation of the measurement model involved the assessment of the discriminant validity of the study constructs. This step specifically showed the extent to which one construct was distinct from other constructs in the structural model (Hair et al., 2019). In this evaluation, the shared variance of the constructs of the model should not be larger than their AVE (Fornell and Larcker, 1981a). In this step, the Fornell–Larcker criterion was employed for this purpose. Table 4 provides the results of this criterion in this study.

**TABLE 4:** Fornell–Larcker criterion.

	Entrepreneurial intention	Innovativeness	Internal locus of control	Need for achievement	Propensity to Take Risks
Entrepreneurial intention	0.838				
Innovativeness	0.454	0.737			
Internal locus of control	0.416	0.496	0.808		
Need for achievement	0.302	0.524	0.504	0.828	
Propensity to take risks	0.247	0.197	0.133	0.094	0.829

*Source: Primary data.*

The Fornell–Larcker criterion, as shown in Table 4, refers to how empirically distinct one construct is from other constructs in the structural model. It also implies that the pooled variance of all model constructs should not be greater than their individual variance (Fornell and Larcker, 1981b).

### The Structural Model

#### Collinearity Issue

Once the measurement model had been evaluated, the next step was to assess the structural model. However, before the structural relationships could be examined, the issue of collinearity had to be addressed to ensure that no bias existed in the regression results. The variance inflation factor (VIF) was employed here to examine collinearity. If the VIF was greater than 5, this would indicate a collinearity issue in the study constructs (Becker et al., 2015). Table 5 presents the findings concerning collinearity.

**TABLE 5:** Collinearity.

	Entrepreneurial intention
**Entrepreneurial intention**	
Innovativeness	1.567
Internal locus of control	1.492
Need for achievement	1.548
Propensity to take risks	1.043

*Source: Primary data.*

Table 5 shows that all values were less than 5, indicating no collinearity.

### Explanatory Power

As Table 5 showed no collinearity in the study constructs, the evaluation of the coefficient of determination (R^2^), which refers to the sum of the influence of exogenous latent variables on the endogenous latent variable, was used to demonstrate the explanatory power of the structural model.

As the result of the R^2^ in Table 6 was greater than 0.25, the explanatory power of the model was considered to not be weak. In other words, the model could explain about 25% of the variance in entrepreneurial intention. In fact, there is no rule of thumb for R^2^ as variations in its result might depend on the discipline and context.

**TABLE 6:** Coefficient of determination (R^2^).

	R square	R square adjusted
Entrepreneurial intention	0.277	0.259

*Source: Primary data.*

### Construct Cross-Validated Redundancy

Table 7 displays the cross-validated redundancy of the constructs. The model of the study had sufficient predictive power because the 1-SSE/SSO values were greater than zero.

**TABLE 7:** Construct cross-validated redundancy.

	SSO	SSE	Q^2^ (= 1-SSE/SSO)
Entrepreneurial intention	660.000	545.908	0.173
Innovativeness	495.000	495.000	
Internal locus of control	330.000	330.000	
Need for achievement	330.000	330.000	
Propensity to take risks	330.000	330.000	

*Source: Primary data.*

**FIGURE 2:**
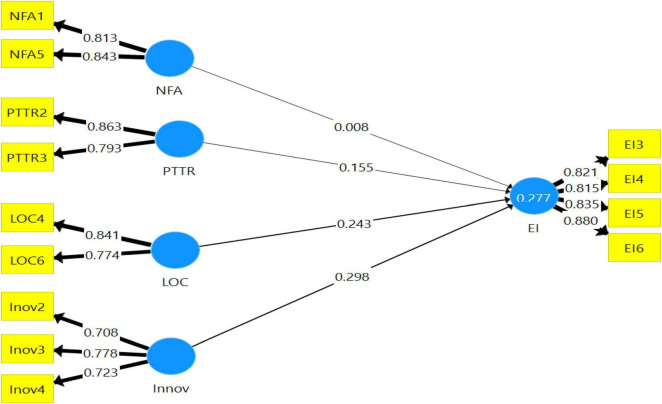
Path coefficients. Source: Primary data.

Figure 2 depicts the results of the structural relationships and their path coefficients.

### Hypothesis Testing

This section demonstrates the bootstrapping procedure, executed with 5,000 resamples, employed to test the hypotheses.

Table 8 shows the results of the path coefficients for the hypothesized relationships of the study. The data in the table reveal that the innovativeness characteristic had a significant positive relationship with the entrepreneurial intention of the students (potential entrepreneurs) (β = 0.298, *p* < 0.10). Likewise, internal locus of control also had a significant positive relationship with the entrepreneurial intention of the students (potential entrepreneurs) (β = 0.243, *p* < 0.10). Furthermore, the propensity to take risks had a positive relationship with the entrepreneurial intention of the students (potential entrepreneurs) as well (β = 0.075, *p* < 0.10). In contrast, the need for achievement had no relationship with the entrepreneurial intention of the students (potential entrepreneurs). Accordingly, H1, H2, and H4 were accepted, whereas H3 was rejected.

**TABLE 8:** Path coefficients.

		β	M	*t*-value	*p*-value	Decision
H1	Innovativeness → entrepreneurial intention	0.298	0.312	3.017	0.003	Accepted
H2	Internal locus of control → entrepreneurial intention	0.243	0.240	2.702	0.007	Accepted
H3	Need for achievement → entrepreneurial intention	0.008	0.019	0.083	0.934	Rejected
H4	Propensity to take risks → entrepreneurial intention	0.155	0.159	1.781	0.075	Accepted

*Source: Primary data.*

## Discussion

This study investigated the influence of selected personality traits—namely, innovativeness, internal locus of control, need for achievement and propensity to take risks. The study generated interesting results. Concerning innovativeness, a significant positive association was found with the entrepreneurial intention of the students (potential entrepreneurs). Indeed, this finding is unsurprising given that more innovative individuals tend to be more open-minded and to think and act innovatively with respect to commercial activities and strategies (Koh, 1996; Shahzad et al., 2021). These individuals are also more adept at solving complex challenges and creating new products and services (Wathanakom et al., 2020). This finding is in line with Law and Breznik (2017), Nasip et al. (2017), Wathanakom et al. (2020), and Shahzad et al. (2021).

The study further examined the influence of internal locus of control on entrepreneurial intention and reported a significant positive result. This is because individuals with high internal locus of control can more easily control and manage their lives and hence have a stronger entrepreneurial intention and become entrepreneurs faster (Vodă and Florea, 2019; Arkorful and Hilton, 2021). The results concerning internal locus of control and entrepreneurial intention are in line with Gurol and Atsan (2006), Lefcourt (2014), Vodă and Florea (2019), and Arkorful and Hilton (2021). With regard to the claim that there is a positive connection between propensity to take risks and entrepreneurial intention, the current study also reported a positive connection with entrepreneurial intention among students (potential entrepreneurs). This is because those individuals with a greater propensity to take risks have higher capacity-building potential, which in turn cultivates a more positive attitude and strengthens self-confidence, both ultimately valuable in venture creation. This finding is in line with Uddin and Bose (2012), Mat et al. (2020), Ndofirepi (2020), Mat et al. (2020), and Shahzad et al. (2021).

Conversely, the need for achievement had no association with entrepreneurial intention. This may be attributable to the young age of the participants—i.e., they may have yet to fully understand the importance of achievement and success in life. This finding is in line with Koh (1996) and Widjaya et al. (2021).

## Implications

As previously recommended in the existing literature, there is a need to continue to investigate the importance of personal traits with regard to entrepreneurial intention among young adults. This study sought to ascertain the impact of specific personal characteristics on the entrepreneurial intentions of Saudi students (potential entrepreneurs). The study yielded intriguing findings that may be of benefit to a variety of stakeholders in Saudi Arabia. First and foremost, the study expands the existing literature by contributing empirical evidence on the relationship between personal traits and entrepreneurial intention.

This research also paved the way for other researchers to continue investigating the same issue by including other personal traits in the analysis as well as moderating and mediating variables in potential conceptual models. The study may also draw the attention of the Saudi government and other official entities to the need to continue to develop necessary training programs and initiatives designed to enhance personal traits among potential young entrepreneurs, particularly those related to developing innovativeness, internal locus of control and propensity to take risks. There is also a need to develop a suitable entrepreneurial ecosystem with appropriate institutional infrastructures that will support Saudi society and culture and encourage entrepreneurial activities.

The Saudi government should also implement public policy programs that encourage entrepreneurial activity by reducing the challenges and barriers faced by potential young entrepreneurs. Furthermore, educational institutions, such as universities and schools, should incorporate the development of personal traits into their curricula and seek to raise awareness about the importance of cultivating such traits for their future endeavors. These educational institutions could work to instill and sustain these personal characteristics in young people, directing them toward entrepreneurial activities and venture creation.

## Conclusion

Personal traits have been identified as key factors influencing entrepreneurial intention in numerous studies. Because of their significance, the present study investigated the ways in which selected personal characteristics—innovativeness, internal locus of control, need for achievement and propensity to take risks—influenced entrepreneurial intention among Saudi students at an applied college. The findings of the study supported all but one of the proposed hypotheses: the hypothesized relationship between the need for achievement and entrepreneurial intention. This study had some limitations, including its small sample size and the restricted context, making it difficult to generalize the findings. Furthermore, no moderation or mediation constructs were considered in the study. It is thus recommended that future studies broaden the scope of research and increase the sample size. The moderating effect of self-confidence on the relationship between other personal characteristics and entrepreneurial intention could also be investigated.

## Data Availability Statement

The raw data supporting the conclusions of this article will be made available by the authors, without undue reservation.

## Author Contributions

Both authors listed have made a substantial, direct, and intellectual contribution to the work, and approved it for publication.

## Conflict of Interest

The authors declare that the research was conducted in the absence of any commercial or financial relationships that could be construed as a potential conflict of interest.

## Publisher’s Note

All claims expressed in this article are solely those of the authors and do not necessarily represent those of their affiliated organizations, or those of the publisher, the editors and the reviewers. Any product that may be evaluated in this article, or claim that may be made by its manufacturer, is not guaranteed or endorsed by the publisher.

## References

[B1] Ajzen I. (1991). The theory of planned behavior. *Org. Behav. Hum. Dec. Proc.* 50 179–211. 10.1016/0749-5978(91)90020-T

[B2] Ali I. Ali M. Badghish S. (2019). Symmetric and Asymmetric Modeling of Entrepreneurial Ecosystem in Developing Entrepreneurial Intentions among Female university Students in Saudi Arabia. *Internat. J. Gend. Entrepr.* 11 435–458. 10.1108/ijge-02-2019-0039

[B3] Aljaghthami E.-N. Noormala A. I. (2016). Saudi Arabia women teachers’ psychological capital towards work engagement. *J. Internat. Bus. Econ. Entrepr.* 1 39–45. 10.24191/jibe.v1i1.14468

[B4] Aljarodi A. (2020). *Female Entrepreneurial Activity in Saudi Arabia : An Empirical Study, Ph.D. thesis.* Bellaterra: Universitat Autonoma de Barcelona,International Doctorate in Entrepreneurship and Management, 2020.

[B5] Al-Jubari I. (2019). College students’ entrepreneurial intention: testing an integrated model of SDT and TPB. *Sage Open* 9 1–15. 10.1177/215824401985346734290901

[B6] Alkahtani N. S. M. S. Delany K. Adow A. H. E. (2020). The Influence of Psychological capital on Workplace Wellbeing and Employee Engagement among Saudi Workforce. *Hum. Soc. Sci. Rev.* 8 233–245. 10.18510/hssr.2020.8522

[B7] Aloulou W. J. (2021). The Influence of Institutional Context on Entrepreneurial Intention: Evidence from the Saudi Young Community. *J. Enterp. Comm.* 2021:19.

[B8] Alshebami A. Al-jubari I. Alyoussef I. Raza M. (2020). Entrepreneurial education as a predicator of community college of Abqaiq students ’ entrepreneur- ial intention. *Manag. Sci. Lett.* 10 3605–3612. 10.5267/j.msl.2020.6.033

[B9] Alshebami A. Seraj A. H. (2021b). The Antecedents of Saving Behavior and Entrepreneurial Intention of Saudi Arabia University Students. *Educ. Sci. Theor. Pract.* 21 67–84. 10.12738/jestp.2021.2.005

[B10] Alshebami A. Seraj A. (2021a). The Impact of Intellectual Capital on the Establishment of Ventures for Saudi Small Entrepreneurs : GEM Data Empirical Scrutiny. *Rev. Internat. Geogr. Educ.* 11 129–142. 10.48047/rigeo.11.08.13

[B11] Alshebami A. S. (2021). The Influence of Psychological Capital on Employees’ Innovative Behavior: mediating Role of Employees’ Innovative Intention and Employees’ Job Satisfaction. *Sage Open* 11:3. 10.1177/21582440211040809

[B12] Arkorful H. Hilton S. K. (2021). Locus of Control and Entrepreneurial Intention: a Study in a Developing Economy. *J. Econ. Adminis. Sci.* 2021:51.

[B13] Becker J.-M. Ringle C. Sarstedt M. Völckner F. (2015). How collinearity affects mixture regression results. *Mark. Lett.* 26 643–659. 10.1007/s11002-014-9299-9

[B14] Bellò B. Mattana V. Loi M. (2018). The Power of Peers A New Look at the Impact of Creativity, Social Context and Self-efficacy on Entrepreneurial Intentions. *Internat. J. Entrepr. Behav. Res.* 24 214–233. 10.1108/ijebr-07-2016-0205

[B15] Bhatti M. AlDoghan M. Saat S. Juhari A. Alshagawi M. (2021). Entrepreneurial Intentions among Women: does Entrepreneurial Training and Education Matters? (Pre- and Post-evaluation of Psychological Attributes and its Effects on Entrepreneurial Intention). *J. Small Bus. Enterp. Dev.* 28 167–184. 10.1108/jsbed-09-2019-0305

[B16] Cai L. Murad M. Ashraf S. F. Naz S. (2021). Impact of dark tetrad personality traits on nascent entrepreneurial behavior : the mediating role of entrepreneurial intention. *Front. Bus. Res. China* 15 1–19. 10.1186/s11782-021-00103-y

[B17] Chew T. C. Tang Y. K. Buck T. (2021). The Interactive Effect of Cultural Values and Government Regulations on Firms ’ Entrepreneurial Orientation. *J. Small Bus. Enterp. Dev.* 2021:228. 10.1108/JSBED-06-2021-0228

[B18] Dinis A. Paco A. Ferreira J. Raposo M. Rodrigues R. (2013). Psychological Characteristics and Entrepreneurial Intentions among Secondary Students. *Educ. Train.* 55 763–780. 10.1108/et-06-2013-0085

[B19] Elnadi M. Gheith M. (2021). Entrepreneurial Ecosystem, Entrepreneurial Self-efficacy, and Entrepreneurial Intention in Higher Education: evidence from Saudi Arabia. *Internat. J. Manag. Educ.* 19 1–12.

[B20] Ferreira J. Raposo M. Rodrigues R. Dinis A. Paco A. (2012). A Model of Entrepreneurial Intention An application of the Psychological and Behavioral Approaches. *J. Small Bus. Enterp. Dev.* 19 424–440. 10.1108/14626001211250144

[B21] Fornell C. Larcker D. F. (1981a). Structural Equation Models With Unobservable Variables and Measurement Error: algebra and Statistics Al. *J. Mark. Res.* 1981 8–382.

[B22] Fornell C. Larcker D. F. (1981b). Evaluating Structural Equation Models with Unobservable Variables and Measurement Error. *J. Mark. Res.* 18 39–50. 10.2307/3151312

[B23] Gurel E. Altinay L. Daniele R. (2010). Tourism Students’ Entrepreneurial Intentions. *Ann. Tour. Res.* 37 646–669. 10.1016/j.annals.2009.12.003

[B24] Gurol Y. Atsan N. (2006). Entrepreneurial Characteristics amongst University Students Some Insights for Entrepreneurship Education and Training in Turkey. *Educ. Train.* 48 25–38. 10.1108/00400910610645716

[B25] Hair J. Hult G. Ringle C. Sarstedt M. (2017). *A Primer on Partial Least Squares Structural Equation Modeling (PLS-SEM) (second).* Thousand Oaks: SAGE Publications Ltd.

[B26] Hair J. Risher J. Sarstedt M. Ringle C. (2019). When to use and how to report the results of PLS-SEM. *Eur. Bus. Rev.* 31 2–24. 10.1108/ebr-11-2018-0203

[B27] Hair J. F. Ringle C. Sarstedt M. (2011). PLS-SEM: Indeed a Silver Bullet. *J. Mark. Theory Pract.* 19 139–152. 10.2753/mtp1069-6679190202

[B28] Ibrahim M. M. S. Amari A. A. (2018). Influence of the Psychological Capital and Perceived Organizational Support on Subjective Career Success: the Mediating Role of Women’s Career Adaptability in the Saudi Context. *Internat. J. Bus. Manag.* 13 1833–8119.

[B29] Jiatong W. Murad M. Bajun F. Tufail M. Mirza F. Rafiq M. (2021). Impact of entrepreneurial education, mindset, and creativity on entrepreneurial intention: mediating role of entrepreneurial self-efficacy. *Front. Psychol.* 12:724440. 10.3389/fpsyg.2021.724440 34497568 PMC8419428

[B30] Khan A. Alsharif N. N. (2019). *SMEs and Vision 2030, Jadwa Investment.* Available online at: http://www.jadwa.com/en (accessed February 25, 2022).

[B31] Koe W.-L. (2016). The Relationship between Individual Entrepreneurial Orientation (IEO) and Entrepreneurial Intention. *J. Glob. Entrepr. Res.* 6:8. 10.1186/s40497-016-0057-8

[B32] Koh H. C. (1996). Testing Hypotheses of Entrepreneurial Characteristics A Study of Hong Kong MBA students. *J. Manag. Psychol.* 11 12–25. 10.1108/02683949610113566

[B33] Krueger N. F. (2000). The Cognitive Infrastructure of Opportunity Emergence. *Entrepr. Theory Pract.* 25 5–23. 10.1177/104225870002400301

[B34] Kusumawijaya I. (2019). The Prediction of Need for Achievement to Generate Entrepreneurial Intention: a Locus of Control Mediation Ida. *Internat. Rev. Manag. Mark.* 9 1–9.

[B35] Law K. Breznik K. (2017). Impacts of innovativeness and attitude on entrepreneurial intention: among engineering and non-engineering students. *Int. J. Technol. Des. Educ.* 27 683–700. 10.1007/s10798-016-9373-0

[B36] Lefcourt H. (2014). *Locus of Control, Current Trends in Theory and Research (second).* Hove: Psychology Press.

[B37] Lewandowska A. Stopa M. Inglot-Brzęk E. (2021). Innovativeness and Entrepreneurship: socioeconomic Remarks On Regional Development In Peripheral Regions. *Econ. Soc.* 14 222–235. 10.14254/2071-789x.2021/14-2/12

[B38] Li C. Murad M. Shahzad F. Aamir M. Khan S. (2020). Entrepreneurial Passion to Entrepreneurial Behavior : role of Entrepreneurial Entrepreneurial Passion to Entrepreneurial Behavior : role of Entrepreneurial Alertness, Entrepreneurial Self-Efficacy and Proactive Personality. *Front. Psychol.* 11 1–20. 10.3389/fpsyg.2020.01611 32973593 PMC7468520

[B39] Liñán F. Chen Y-W (2009). Development and Cross-Cultural Application of a Specific Instrument to Measure Entrepreneurial Intentions. *Entrepr. Theory Pract.* 33 593–617. 10.1111/j.1540-6520.2009.00318.x

[B40] Marton G. Monzani D. Vergani L. Pizzoli S. Pravettoni G. (2021). How to Measure Propensity to Take Risks in the Italian Context: the Italian Validation of the Risk Propensity Scale. *Psycholog. Rep.* 0 1–15. 10.1177/00332941211054777 34879777

[B41] Mat Z. Yusoff M. Zainol F. Afthanorhan A. (2020). Risk-taking propensity & personality of women entrepreneurs in Malaysia. *J. Crit. Rev.* 7 1214–1221.

[B42] McClelland D. (1961). *The Achieving Society.* New York, NY: Princeton, N.J. Van Nostrand, 1961.

[B43] Mueller S. L. Thomas A. S. (2000). Culture and entrepreneurial potential: A nine country study of locus of control and innovativeness. *J. Bus. Vent.* 16 51–75. 10.1016/S0883-9026(99)00039-7

[B44] Nasip S. Amirul S. Jr S. (2017). Psychological Characteristics and Entrepreneurial Intention A Study among University Students in North Borneo, Malaysia. *Educ. Train.* 59 825–840. 10.1108/et-10-2015-0092

[B45] Naushad M. (2018). A study on the Antecedents of Entrepreneurial Intentions among Saudi Students. *Entrepr. Sust. Center* 5 600–617. 10.9770/jesi.2018.5.3(14)

[B46] Ndofirepi T. M. (2020). Relationship between entrepreneurship education and entrepreneurial goal intentions: psychological traits as mediators. *J. Innov. Entrep.* 9:115. 10.1186/s13731-020-0115-x

[B47] Rauch A. Frese M. (2007). Let’s put the person back into entrepreneurship research: A meta-analysis on the relationship between business owners’ personality traits, business creation, and success. *Eur. J. Work Org. Psychol. ISSN* 16 353–385. 10.1080/13594320701595438

[B48] Robinson P. B. Huefner J. C. Hunt H. K. (1991). Entrepreneurial Research on Student Subjects Does Not Generalize to Real World Entrepreneurs. *J. Small Bus. Manag.* 29 42–50.

[B49] Roomi M. Kelley D. Coduras A. (2021). *Kingdom of Saudi Arabia National Report 2020–2021.* Santiago: Global Entrepreneurship Monitor Report (GEM).

[B50] Rotter J. (1966). Generalized Expectancies for Internal versus External Control of Reinforcement. *Psychol. Monogr. Gen. Appl.* 80 1–28. 10.1037/h0092976 5340840

[B51] Schaper M. Volery T. Weber P. Lewis K. (2010). Entrepreneurship and Small Business (3rd Asia-P). *Asia-Pacific edit.* 20 2010.

[B52] Shahzad M. Khan K. Saleem S. Rashid T. (2021). What Factors Affect the Entrepreneurial Intention to Start-Ups? The Role of Entrepreneurial Skills, Propensity to Take Risks, and Innovativeness in Open Business Models. *J. Open Innov.* 7 1–23.

[B53] Sharahiley S. M. (2020). Examining Entrepreneurial Intention of the Saudi Arabia’s University Students. *Glob. J. Flexib. Syst. Manag.* 21 67–84.

[B54] Shaver K. G. Scott L. R. (1992). Person, Process, Choice: the Psychology of New Venture Creation. *Entrepr. Theory Prac.* 16 23–46. 10.1177/104225879201600204

[B55] Staniewski M. W. Nowacki R. Awruk K. (2016). Entrepreneurship and innovativeness of small and medium-sized construction enterprises. *Internat. Entrepr. Manag. J.* 12 861–877. 10.1007/s11365-016-0385-8

[B56] Tong X. F. Tong D. Y. K. Loy L. C. (2011). Factors Influencing Entrepreneurial Intention Among University Students. *Internat. J. Soc. Sci. Hum. Stud.* 3 487–496.

[B57] Uddin M. R. Bose T. K. (2012). Determinants of Entrepreneurial Intention of Business Students in Bangladesh. *Internat. J. Bus. Manag.* 7:128. 10.5539/ijbm.v7n24p128

[B58] Vodă A. I. Florea N. (2019). Impact of Personality Traits and Entrepreneurship Education on Entrepreneurial Intentions of Business and Engineering Students. *Sustainab.* 11:1192. 10.3390/su11041192

[B59] Wathanakom N. Khlaisang J. Songkram N. (2020). The study of the Causal Relationship between Innovativeness and Entrepreneurial Intention among Undergraduate Students. *J. Innov. Entrepr.* 9:5. 10.1186/s13731-020-00125-5

[B60] Widjaya O. Budiono H. Wiyanto H. Fortunata F. (2021). The Effect of Locus of Control, Need for Achievement, Risk Tolerance, and Entrepreneurial Alertness on the Entrepreneurial Intention. *Adv. Soc. Sci. Educ. Hum. Res.* 570 177–184.

[B61] Yaser A-M. Abdulrab M. Alwaheeb M. A. Alshammari N. (2020). Factors Impacting Entrepreneurial Intentions among University Students in Saudi Arabia: Testing an Integrated Model of TPB and EO. *Educ. Train.* 62 779–803. 10.1108/et-04-2020-0096

[B62] Zhao H. Wibowo A. (2021). Entrepreneurship Resilience: can Psychological Traits of Entrepreneurial Intention Support Overcoming Entrepreneurial Failure? *Front. Psychol.* 12 1–12. 10.3389/fpsyg.2021.707803 34594271 PMC8476748

